# Bearing My Heart: The Role of Extracellular Matrix on Cardiac Development, Homeostasis, and Injury Response

**DOI:** 10.3389/fcell.2020.621644

**Published:** 2021-01-12

**Authors:** Ana Catarina Silva, Cassilda Pereira, Ana Catarina R. G. Fonseca, Perpétua Pinto-do-Ó, Diana S. Nascimento

**Affiliations:** ^1^i3S – Instituto de Investigação e Inovação em Saúde, Universidade do Porto, Porto, Portugal; ^2^INEB – Instituto Nacional de Engenharia Biomédica, Universidade do Porto, Porto, Portugal; ^3^Gladstone Institutes, San Francisco, CA, United States; ^4^ICBAS – Instituto de Ciências Biomédicas Abel Salazar, Universidade do Porto, Porto, Portugal

**Keywords:** heart, extracellular matrix, cardiac ontogeny, cardiovascular diseases, decellularization, fibrosis, regeneration

## Abstract

The extracellular matrix (ECM) is an essential component of the heart that imparts fundamental cellular processes during organ development and homeostasis. Most cardiovascular diseases involve severe remodeling of the ECM, culminating in the formation of fibrotic tissue that is deleterious to organ function. Treatment schemes effective at managing fibrosis and promoting physiological ECM repair are not yet in reach. Of note, the composition of the cardiac ECM changes significantly in a short period after birth, concurrent with the loss of the regenerative capacity of the heart. This highlights the importance of understanding ECM composition and function headed for the development of more efficient therapies. In this review, we explore the impact of ECM alterations, throughout heart ontogeny and disease, on cardiac cells and debate available approaches to deeper insights on cell–ECM interactions, toward the design of new regenerative therapies.

## Introduction

The heart is one of the least regenerative organ systems in mammals, which partially explains the high mortality and morbidity rates of cardiovascular diseases (Virani et al., 2020). Long considered a post-mitotic organ and well-illustrated by deficient myocardium renewal capacity, it is nowadays known to retain cardiomyocyte turnover throughout life (Beltrami et al., 2001; Bergmann et al., 2009; Bergmann and Jovinge, 2014; Lázár et al., 2017) although at levels incompatible with the restoration of tissue function in disease or after injury (Porrello et al., 2011). For example, cardiomyocytes lost after myocardial infarction (MI) are not replaced by new muscle, and instead, nonfunctional fibrotic tissue is deposited in the affected region (replacement fibrosis).

Effective regeneration of the mammalian heart is observed only during fetal–neonatal stages and requires triggering the proliferation of preexisting cardiomyocytes (Robledo, 1956; Herdrich et al., 2010; Porrello et al., 2011; Uygur and Lee, 2016; Sampaio-Pinto et al., 2018). This regenerative capacity falls abruptly in the first days after birth, which coincides with the final phase of cardiomyocyte maturation and concomitant cessation of proliferative activity (Quaini et al., 2002; Porrello et al., 2011; Notari et al., 2018; Sampaio-Pinto et al., 2018; Ye et al., 2018; Zhu et al., 2018). Despite this time limitation, the neonatal regenerative capacity has been widely dissected toward identification of pro-regenerative mechanisms. However, most studies are yet centered on the cellular compartment of the heart, overlooking the role of the extracellular matrix (ECM) in this response (Bassat et al., 2017).

Herein, the relevance of the ECM for healthy and diseased hearts and the importance of addressing the ECM for new cutting-edge regenerative therapies will be revisited and discussed.

## Cardiac ECM

The ECM constitutes a complex network of fibrillary (fibrillar collagens) and non-fibrillary (composed by the basement membrane, proteoglycans, and glycoproteins) components within the extracellular space that have both signaling and structural functions (Chute et al., 2019). New proteomic approaches have revealed that 90% of cardiac ECM is composed of 10 different proteins, from which serum albumin, collagens (collagens I, III, and IV), non-collagenous glycoproteins [fibronectin (FN) and laminin], proteoglycans, glucosaminoglycans (GAGs), and elastins are the most common (Lindsey et al., 2018). The fibrillar collagenous matrix comprises essentially type I (over 80%) and type III (over 10%) collagens (Weber, 1989) anchored to the myocardial cell basement membranes through collagen type IV and FN (Bashey et al., 1992; McCurdy et al., 2010). In addition, ECM works as a reservoir of anchored growth factors, cytokines, chemokines, proteases [e.g., matrix metallopeptidases (MMPs)], proteases inhibitors [e.g., tissue inhibitors of metalloproteinases (TIMPs)], and noncoding RNAs such as microRNAs (miRNAs) (Hynes, 2009; Jourdan-Lesaux et al., 2010; Fan et al., 2014).

Spatially, ECM is organized into two main regions, the basement membrane/pericellular matrix and interstitial matrix. The basement membrane/pericellular matrix constitutes a tissue specialized network of ECM molecules that involve each cell, promoting cell polarity and function (e.g., differentiation and migration) via cell surface receptors, such as integrins, through an outside–in signaling (Corda et al., 2000; LeBleu et al., 2007; Valiente-Alandi et al., 2016). This ECM compartment is mainly composed of FN, collagen IV, laminin, procollagens, hyaluronic acid (HA), and proteoglycans (Chang et al., 2016). As for the interstitial matrix, ECM molecules, such as collagens I and III, granting structural and mechanical support to the tissue, are main constituents.

The cell modulatory nature of the extracellular microenvironment results from a continuous remodeling of ECM composition and structural rearrangement, but also by the formation of bioactive peptides, known as matrikines, via enzymatic degradation of the ECM macromolecules (Maquart et al., 1999, 2005). These alterations affect cell function but also promote ECM remodeling in a feedback loop. ECM remodeling occurs in waves, a consequence of the tight control between synthesis and degradation, and generates active extracellular niches that regulate different cellular responses, namely, proliferation, migration, cell fate decisions, and even cell death. Cardiac fibroblasts (cFBs) are majorly responsible for ECM production and remodeling under homeostatic and pathological conditions, but other cells also contribute to the synthesis of ECM, particularly to the basement membrane, such as endothelial cells, smooth muscle cells, and cardiomyocytes (Aggeler, 1988; Pelouch et al., 1993; Corda et al., 2000; Anderson and Hinds, 2012; Bax et al., 2019).

Several transmembrane cell surface molecules such as CD44 and integrins, among others, mediate the bidirectional communication between cells and their environment (Valiente-Alandi et al., 2016). Integrin-mediated adhesions are the most frequent and well-described cell–ECM interactions (Howard and Baudino, 2014). Integrins are a large family of transmembrane receptors composed by α and β subunits and splice variants with different ligand specificities that undergo several conformational changes that impact integrin–ECM affinity (Ross, 2004; Askari et al., 2009). The integrin subunits expressed on cardiomyocytes throughout heart development and their ECM ligands have been extensively reviewed elsewhere (Ross, 2004). Integrin receptors interact directly or indirectly with different molecules, such as focal adhesion kinases, and with actin cytoskeleton filaments that, upon activation, trigger several signaling cascades regulating cellular processes (Askari et al., 2009). ECM–integrin–cytoskeleton linkage also mediates mechanotransduction signaling (Sun et al., 2016; Jansen et al., 2017). The mechanism that links integrin signaling with well-recognized mechanotransduction pathways, e.g., YAP/TAZ [Yes-associated protein (YAP) and transcriptional coactivator with PDZ-binding motif (TAZ)], is not completely understood, but the possibility of a direct correlation between integrins and YAP/TAZ signaling has recently emerged (Elbediwy et al., 2016; Chakraborty et al., 2017; Sabra et al., 2017; Martino et al., 2018). Integrins and actin filaments have been demonstrated to interact directly or indirectly with protein adaptors, such as vinculin, talin, and α-actinin (Schwartz, 2010). Indeed, transduction of mechanical changes observed on intracellular (formation of stress fibers) and extracellular spaces (ECM stiffness) is an important regulator of cardiomyocyte behavior through the regulation of the expression of mechanosensitive genes such *egr-1*, *iex-1*, and *c-fos* (Engler et al., 2008; Jaalouk and Lammerding, 2009; Martino et al., 2018).

Heart morphogenesis, maturation, and pathophysiology result from intrinsic factors related to the cellular transcriptional landscape, but also extrinsic cues from the ECM. Comprehension of ECM dynamics and cell modulatory properties is crucial for the establishment of *in vitro* culture systems that recapitulate *in vivo*-like microenvironments, but also for development of more effective heart therapies.

## Role of ECM in Cardiac Ontogeny

### Embryonic and Fetal Heart

Throughout heart development, the ECM supports a tight spatiotemporal regulation of different cellular processes and progresses from a high hydrated gel rich in morphogenic molecules to a structurally defined collagen network poor in morphogens.

**FIGURE 1:**
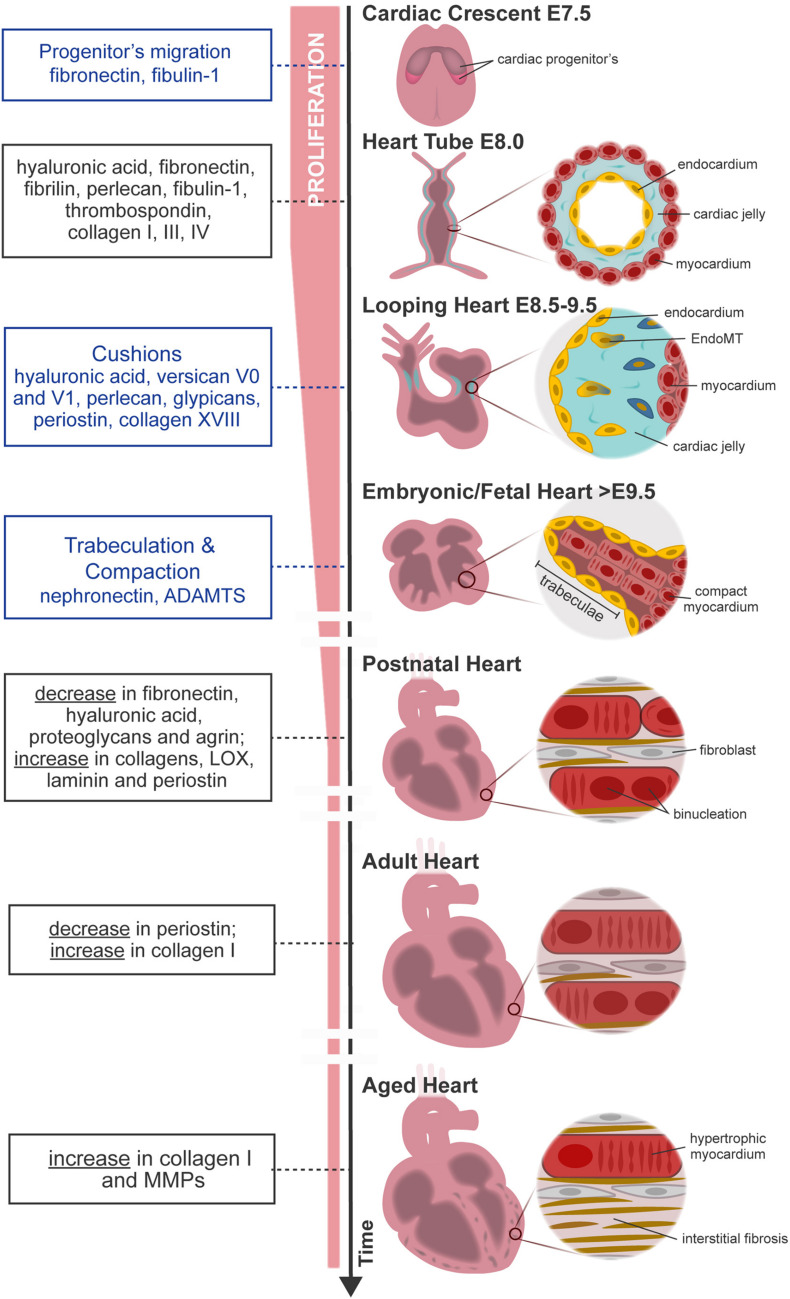
Overview of mouse heart development, maturation, and aging. FN from primitive ECM paves the way for the migration of cardiac progenitor cells **(cardiac crescent)** to the embryo midline. Upon fusion, cardiac progenitor cells form the **heart tube**. The latter is constituted by two cell layers—the myocardium (outer layer) and the endocardium (inner layer)—separated by an amorphous matrix known as the cardiac jelly. The heart starts looping **(looping heart)** toward the formation of a four-chambered organ. In parallel, endocardial cells invade the cardiac cushion, that is, an extensive accumulation of cardiac jelly at primitive valve structures, and undergo EndoMT, forming valve tissue cells. The heart evolves, and the size of the myocardium increases while cardiomyocytes proliferate and mature at the compact and trabecular layers, respectively. Compaction and trabeculation are regulated by the transient expression of nephronectin and by the enzymatic degradation of the versican promoted by ADAMTs **(fetal heart)**. After birth, the ECM undergoes extensive remodeling characterized by a decrease in hyaluronic acid, FN, and proteoglycans. At the same time, cardiomyocytes cease proliferation and finalize maturation, acquiring robust sarcomeres and a rod-shaped morphology **(postnatal–adult heart)**. Aging contributes to functional impairment by the loss of cardiomyocytes and formation of fibrotic tissue **(aged heart)**. Blue box, specific morphological events regulated by the ECM; black box, variations on ECM composition throughout ontogeny.

At E7.5, myocardial precursors, also known as primary and secondary heart fields, migrate to the midline of the embryo forming the heart tube (Figure 1, cardiac crescent). A primitive heart ECM derived from endoderm, mainly composed by chondroitin sulfate, collagens I and IV, laminin, fibulin, fibrillin, and FN, orchestrates the migration of these precursors (Little and Rongish, 1995; Lockhart et al., 2011). Among these molecules, FN promotes the migration of myocardial precursors toward the embryo midline by temporal modulation of cell adhesion and polarity. Consequently, FN mutation leads to aberrant formation of the heart such as cardia bifida, resulting in embryonic lethality (Linask and Lash, 1988; George et al., 1993; Trinh and Stainier, 2004; Mittal et al., 2013).

At E8.0, the heart has a tubular shape and is composed of two cell layers—the myocardium (outer layer) and the endocardium (inner layer)—separated by an amorphous matrix-denominated cardiac jelly (Figure 1, heart tube). The cardiac jelly consists of a network of ECM molecules enriched in HA; collagens I, III, and IV; laminin; FN; fibrillin; perlecan; fibulin-1; and thrombospondin (TSP) (Little and Rongish, 1995; Männer and Yelbuz, 2019). The heart expands by the contribution of second heart field (SHF) cells and undergoes a series of looping events at E8.5 (Figure 1, looping heart). Although mechanisms behind heart looping are not completely understood, HA is one of the most abundant molecules in the cardiac jelly. While the removal of HA by enzymatic degradation does not affect heart looping progression, the absence of HA leads to extensive alterations in heart tissue hemodynamics (Baldwin et al., 1994; Grandoch et al., 2018; Petz et al., 2019). In addition, ECM has a determinant function on the morphogenesis of specific heart substructures such as trabeculae, valve formation, atrial and ventricular septation, and outflow tract remodeling.

#### Myocardial Trabeculation and Compaction

Trabeculae formation is initiated at day E8.0 of mouse development with the sprouting of the endocardium toward the myocardium. At this stage, the myocardium is multilayered and presents a compact and discontinued (clusters) layer of cardiomyocytes (premature trabeculae cardiomyocytes). At E8.5, endocardial sprouting develops through the cardiac jelly, forming distinct columns that anchor with the compact myocardium—the endocardium touchdowns. Endocardial ridges are formed between the touchdowns, creating domes enriched in HA and FN, and clusters of cardiomyocytes, forming the trabecular units. The cardiomyocytes at the trabecular units organize in a radial disposition and grow in a radial fashion (trabecular extension), peaking around E14.5, concurrent with a progressive reduction of ECM content. Endocardial sprouting and touchdown are regulated by Notch signaling by promoting the expression of several ECM proteases, such as *Adamts1*, *Mmp2*, and *Hyal2* (Table 1; Del Monte-Nieto et al., 2018). The abrogation or overexpression of NOTCH1 signaling has been demonstrated to cause ventricular dysplasia and trabecular defects or ventricular hypertrabeculation, respectively (Grego-Bessa et al., 2007; Chen et al., 2013; Zhao et al., 2014; D’Amato et al., 2016). On the other hand, neuregulin-1 signaling, a downstream target of Notch, promotes the synthesis of ECM components necessary for trabecular growth (Table 1; Passer et al., 2016; Del Monte-Nieto et al., 2018). Thus, trabeculation of the heart is a process dependent not only on ECM synthesis but also on spatial and temporal regulation of ECM degradation (Stankunas et al., 2008; Lockhart et al., 2011; Del Monte-Nieto et al., 2018). Myocardial trabeculation and compaction are two fundamental processes for proper cardiac chamber maturation. Both processes depend on Notch signaling and entail the degradation of the cardiac jelly for proper heart morphogenesis (Sandireddy et al., 2019). In particular, ventricular compaction has been demonstrated to be regulated by other molecules, such as *Slc39a8* zinc transporter and Sema3E/plexinD1 signaling, which positively regulates several ECM proteases from the ADAMTS family (*Adamts1,5,7,17*) (Table 1; Lin et al., 2018; Sandireddy et al., 2019). ADAMTS9 has been also pointed as essential to myocardial compaction by promoting versican degradation as demonstrated by ADAMTS9 haploinsufficient mice that develop abnormal projections and a “spongy” ventricular wall, resembling human hearts with left ventricular non-compaction congenital cardiomyopathy (Table 1; Kern et al., 2010; Lockhart et al., 2011; Sarma, 2011).

**TABLE 1:** Summary table of the signaling pathways that regulate ECM dynamics and associated cell function.

Heart ontogeny/disease	Heart structures	Signaling pathways	ECM remodeling	Cellular impact	References
Embryonic/fetal heart development	Trabeculae	Neuregulin 1	ECM synthesis: • HA • Fibronectin	Polarized cardiomyocyte division	Passer et al., 2016; Del Monte-Nieto et al., 2018
		Notch 1	ECM proteolysis: • ADAMTS1 • MMP2 • HYAL2		Del Monte-Nieto et al., 2018
	Myocardial compaction	Notch 1 Slc39a8 Sema3E/plexinD1	ECM proteolysis: • ADAMTS1,5,7,9,17 • MMP2 • HYAL2		Del Monte-Nieto et al., 2018; Lin et al., 2018; Sandireddy et al., 2019
	Atrioventricular and valve cushions	BMP2	ECM synthesis: • HA • Versican	Cardiac cushion mesenchyme migration	Inai et al., 2013
		BMP4 (inhibition)*	Nephronectin ECM reduced proteolysis: • HAS2	Restriction of atrioventricular channel differentiation and cardiac jelly swelling	Patra et al., 2011
		TGF-β3	ECM synthesis: • Periostin • Collagen 1	Differentiation of the cushion mesenchyme into fibroblasts	Norris et al., 2009
	Outflow tract remodeling	SMAD4	ECM proteolysis: • MT1-MMP	NCC migration	Jia et al., 2007
	Myocardium	β1-integrin*	Fibronectin	Cardiomyocyte proliferation	Ieda et al., 2009
		YAP/TAZ* ERK*	Soft ECM Agrin		Snider et al., 2008; von Gise et al., 2012; Bassat et al., 2017
Postnatal–adult heart	Myocardium	YAP/TAZ downregulation*	Stiff ECM	Inhibition of cardiomyocyte differentiation	von Gise et al., 2012
Adult heart ischemia	Epicardium, myocardium	TGF-β/Smad3 Hippo downregulation	ECM synthesis: • Periostin • Collagen 1	Epicardial cells EMT into fibroblast-like cells Fibroblast activation (myofibroblasts)	Liu et al., 2015; Piersma et al., 2015; Travers et al., 2016; Ramjee et al., 2017

*ECM downstream signaling pathways.

#### Atrioventricular and Outflow Tract Cushions

Atrioventricular (AV) and outflow tract cushions form by the differentiation of the local endocardium that, around E9.5, undergoes endocardial-to-mesenchymal transition (EndoMT), invading the neighboring cardiac jelly deposits (Figure 1, looping heart). The cardiac cushion ECM is enriched in HA and proteoglycans (versicans V0 and V1, perlecan, and glypicans) which confer these regions the consistency of a hydrated gel (Baldwin et al., 1994; Lockhart et al., 2011). Other ECM components are also identified on these acellular structures such as FN, collagens, laminin, nephronectin, tenascin-C (TNC), vitronectin, fibulin-1, fibulin-2, fibrillin, and enzymes (chondroitin-6-*O*-sulfotransferase-1 and chondroitin-6-*O*-sulfotransferase-14) (Patra et al., 2011). The content of HA in cardiac cushions is crucial for correct valve formation. Both the impairment and overproduction of HA result in valve malformations and the development of congenital defects (Baldwin et al., 1994; Petz et al., 2019). Bone morphogenic protein (BMP) and transforming growth factor β (TGF-β) signaling are major regulators of heart morphogenesis in mice and avians, including AV septation by modulating cardiac cushion ECM composition, cellular invasion, and differentiation (Ma et al., 2005; Jiao et al., 2006; Prados et al., 2018). Specifically, BMP2 signaling has been implicated in HA and versican production by the chick cardiac cushion mesenchyme promoting its migration *in vitro* (Inai et al., 2013). On the other hand, studies in zebrafish suggested that nephronectin, an ECM component of the cardiac cushions, inhibits BMP4-Has2 signaling restricting AV channel differentiation and cardiac jelly swelling (Patra et al., 2011). TGF-β3 signaling further contributes to the AV valve maturation in mice by promoting the differentiation of the cushion mesenchyme into fibroblasts through the expression of periostin and collagen I production (Table 1; Norris et al., 2009; Lockhart et al., 2011). Periostin-knockout mice (Kii et al., 2006) and Peri^lacZ^-null mice (Rios et al., 2005) exhibit both valve and septal defects (Snider et al., 2008; Lockhart et al., 2011). Collagen XVIII, a non-fibrillar form of collagen, is also expressed during early AV cushion development (Carvalhaes et al., 2006). Col18a1 knockdown leads to thickening of the endothelial basement membrane surrounding the AV valves (Utriainen et al., 2004; Lockhart et al., 2011) with no compromise of the heart function, despite causing hydrocephalus and decreased kidney filtration capacity (Utriainen et al., 2004; Hamano et al., 2010). While periostin and collagen XVIII can be detected in early heart development in the AV and outflow tract cushions and throughout the ventricular wall, as the heart matures, they become confined to specific areas, namely, the valves (Lockhart et al., 2011; González-González and Alonso, 2018).

#### Outflow Tract Development and Remodeling

Outflow tract development is a process that results from the remodeling of the vasculature and the migration of distinct cell types, endothelial/endocardial cells and cardiac neural crest cells (NCCs). Endocardial cells undergo epithelial-to-mesenchymal transition (EMT), invading the ECM-enriched cardiac cushions with the NCCs migrating to the nascent aortopulmonary septum and outflow tract cushion (Jiang et al., 2000). Smad signaling has been shown to impact NCC migration by regulating several vasculature/remodeling [e.g., *Id(1–4)*] and ECM organization-related (e.g., MMPs) genes in mice (Jia et al., 2007; Moskowitz et al., 2011). Specifically, *Smad4* inactivation in NCCs induce a reduction of *Id* gene expression, which negatively impacts ECM proteinase MT1–MMP expression. Similar observations were reported upon *Id1/Id3* inactivation studies. The reduction of MT1–MMP expression affects migration of NCCs, as it translates in accumulation of ECM along the route of OFT caudal movement (Table 1; Jia et al., 2007).

Cardiomyocyte proliferation during fetal development is responsive to the surrounding embedding ECM. Ieda et al. (2009) demonstrated that ECM secreted by embryonic cFB at E12.5 favors cardiomyocyte proliferation more than adult ECM. ECM molecules secreted by embryonic cFB such as FN, TNC, hyaluronan, and proteoglycan link protein 1 have been shown to promote different cellular responses. Thus, while FN favored cardiomyocyte proliferation via β1-integrin signaling, hyaluronan enhanced cell adhesion in an integrin-independent manner (Table 1; Ieda et al., 2009). A similar mitogenic effect of FN has also been reported by Williams et al. (2014) while culturing cardiomyocytes on tissue culture plates coated with enzymatically digested fetal (E18–E19) heart tissue-derived ECM.

Even though the ECM qualitatively remains the same until birth, alterations of ECM molecule abundance and arrangement within the myocardium are observed. For example, laminin networks evolve from a punctuated patch-like deposition in the fetal heart (E11.5–E15) to a more extensive deposition in the developing basal membrane of cardiomyocytes in the neonatal heart and then to a contiguous layer along the basal membrane in the adult heart (Price et al., 1992; Yang et al., 2015). Laminin modulates cell adhesion by interacting with cell receptors such as integrins and forming a transmembrane link to the cytoskeleton via dystroglycan and dystrophin (Henry and Campbell, 1996; Okada et al., 2016). Deficiency on laminin expression results in muscular dystrophies and dilated cardiomyopathy at birth or early childhood, associated with metabolic deficiencies (Cox and Kunkel, 1997; Oliviéro et al., 2000; Yap et al., 2019).

The relevance of the ECM in heart development has been recently strengthened by the generation of three-dimensional (3D) mouse heart organoids *in vitro* in the presence of the laminin–entactin (LN/ET) complex and exogenous fibroblast growth factor 4 (FGF4). These organoids formed from self-organizing embryoid bodies, resulting in structures with atrium- and ventricle-like contractile structures (Lee et al., 2020).

### Postnatal Heart

After birth, the heart undergoes severe alterations at the cellular and extracellular levels to adapt to physiological requirements of the growing body. The ECM is largely remodeled, involving a decline in the abundance of ECM molecules serving as morphogenic cues such as FN, HA, and proteoglycans, along with a concomitant increase in structural molecules, such as collagens I and III and laminin (Kim et al., 1999; Ieda et al., 2009; Williams et al., 2014). These alterations result in a more structured ECM that confines each cardiomyocyte individually, resembling a honeycomb-like organization (Figure 1; Robinson et al., 1988; Pelouch et al., 1993). These alterations coincide with the timing when the regenerative capacity of the heart ceases. Hence, one can argue that alterations at the ECM may be involved in the transition from a regenerative period to a reparative period. In agreement with this perspective, P1 but not P7 cardiac ECM fragments are able to stimulate cell cycle activity of neonatal cardiomyocytes. An elegant study by Notari et al. (2018) demonstrated that pharmacological inhibition of lysyl oxidase (LOX), an ECM cross-linking enzyme using 3-aminopropionitrile, a LOX inhibitor previously described to reduce lung ECM stiffness in newborn mice (Mammoto et al., 2013), could rescue the regenerative capacity of P3 hearts. Similar evidences were observed *in vitro*, wherein Yahalom-Ronen et al. (2015) demonstrated that culture of neonatal cardiomyocytes in rigid surfaces leads to enhanced myofibrillar organization and facilitates karyokinesis. Conversely, compliant surfaces promoted cardiomyocyte rounding, sarcomere disorganization, and cytokinesis. These findings demonstrate that the increase in ECM stiffness around birth might dictate the transition from a regenerative period to a reparative period, strengthening the relevance of the regulatory role of ECM mechanical properties.

YAP is a downstream effector of the Hippo pathway, a well-conserved mechanotransduction pathway on mammals with an important role during embryo development in the regulation of proper organ size. In the heart, YAP/TAZ activity has been mainly implicated in embryonic heart development, in postnatal growth, and in response to injury by promoting cardiomyocyte proliferation through the activation of cell cycle-related genes, such as *Ccna2*, *Ccnb1*, *Cdc2*, *Aurka*, *Aurkb*, and *Cdc25b* (von Gise et al., 2012; Xin et al., 2013; Mosqueira et al., 2014; Singh et al., 2016). The decline of cardiomyocyte proliferation observed post birth seems to correlate with a reduction in YAP expression and increase of YAP phosphorylation (inactivation) observed with aging (Table 1; von Gise et al., 2012). Deletion of *Yap* in the heart hampers neonatal regeneration at P2 and elicits a fibrotic response similar to what is observed in older animals. In fact, overexpression of a constitutively active form of YAP in the heart of 4-week-old mice enhances cardiac function after MI. These studies collectively show that biomechanical alterations at the ECM around birth may influence cardiomyocyte cycling activity and subsequently impact the regenerative capacity of the heart.

### Aging Heart

Cellular aging is characterized by an accumulation of defective molecules and organelles at the cytoplasm and decline of reparative mechanisms. Thus, with age, cardiomyocytes accumulate dysfunctional mitochondria, oxidized proteins such as advanced glycation end products (AGE), and lipofuscin particles, denoted as “cellular garbage” (Kilhovd et al., 1999; Terman et al., 2008). These age-related alterations are progressively deleterious, resulting in a decrease in the number of cardiomyocytes and subsequent pathological hypertrophy of the remaining cardiomyocytes, inflammation, and gradual development of cardiac fibrosis (Bernhard and Laufer, 2008).

Heterochronic parabiosis studies suggest the participation of the extracellular milieu on cardiomyocyte aging by demonstrating that systemic factors impact age-related cardiomyocyte hypertrophy (Loffredo et al., 2013). Hypertrophic cardiomyocytes have a higher demand on oxygen and energy, creating a low-oxygen environment with consequent free radical production and cellular damage, as reviewed in Meschiari et al. (2017). To compensate for the progressive cardiomyocyte loss, the ECM content increases, in particular collagen I (Figure 1; Bernhard and Laufer, 2008; Spadaccio et al., 2015; Horn and Trafford, 2016; Meschiari et al., 2017). Along with the increased collagen deposition and cross-linking, the ECM degradation capacity also augments through the production of MMPs, predominantly MMP9 (Meschiari et al., 2017). Increased MMP activity also attenuates angiogenic activity, contributing to the formation of a deleterious hypoxic environment (Yabluchanskiy et al., 2014).

The excessive accumulation of ECM and imbalance on ECM degradation lead to tissue scarring and cardiac dysfunction. Interstitial fibrosis has a detrimental effect on myocardial function by interfering with cardiomyocyte electrical coupling. The latter is characterized by an accumulation of collagen that separates cardiomyocytes, expediting the emergence of arrhythmogenic events and, in worst cases, sudden cardiac death (Stein et al., 2008; Nguyen et al., 2014).

## ECM in Disease/Fibrotic Heart

Contrary to the regenerative response observed in neonates, the adult heart responds to an insult largely through the development of cardiac fibrosis. While at the start of the reparative process some ECM molecules secreted are similar to those seen in the regenerative response [e.g., FN (Konstandin et al., 2013) and TNC (Kasprzycka et al., 2015)], once cardiomyocytes have exited the cell cycle, upregulation of these ECM constituents is no longer enough, by itself, to induce proliferation. The result is the net accumulation of a collagen-rich ECM in the myocardium and subsequent formation of a stiff scar (Ieda et al., 2009; Hortells et al., 2019). Differences in cell surface receptor expression during development may influence cell response to injury and explain the shift from regeneration to repair. Integrin subunits, for example, are known to vary temporally, by cell type and with disease (Israeli-Rosenberg et al., 2014). A unique integrin profile can be observed in myocytes vs. fibroblasts or endothelial cells, in fetal vs. neonatal or adult myocytes, and in normal vs. pathological hearts (e.g., normal vs. failing or post-MI tissue). In cardiomyocytes, the integrin heterodimers most highly expressed are α1β1, α5β1, and α7β1, predominantly collagen-, FN-, and laminin-binding receptors, respectively (Israeli-Rosenberg et al., 2014). While the α5 subunit is prevalent in fetal and neonatal cardiomyocytes, α7 replaces α5 at the onset of postnatal development and becomes the main subunit detected in mature adult cardiomyocytes (Brancaccio et al., 1998; Israeli-Rosenberg et al., 2014).

### Cardiac Fibrosis as a Response to Injury

Cardiac fibrosis can be reactive, in response to chronic stress (such as inflammation, pressure overload, and aging) without involving cardiomyocyte death, or reparative, when replacing lost cardiomyocytes as observed during MI (Kong et al., 2014). Several other conditions can result in progressive cardiac fibrosis such as hypertrophic cardiomyopathy, toxic insults (e.g., alcohol and anthracyclines), and metabolic disturbances such as diabetes and obesity, as reviewed in Kong et al. (2014). Regardless of the pathological trigger, excessive fibrosis in the myocardium may have a variety of deleterious consequences (Berk et al., 2007). In fact, clinical evidence correlates adverse outcomes in patients with heart failure with increased and stiffer cardiac ECM. Patients with heart failure with preserved ejection fraction (HFpEF) show an expansion of the interstitial ECM network, associated with coronary microvascular rarefaction and inflammatory activation, as reviewed by Paulus and Tschöpe (2013) and Mohammed et al. (2015).

The fibrotic remodeling of the heart results from the relative contribution of several cell types either by directly producing matrix proteins (fibroblasts) or by indirectly secreting fibrogenic mediators (macrophages, mast cells, lymphocytes, cardiomyocytes, and vascular cells). Common to all conditions associated with cardiac fibrosis, fibroblast transdifferentiation into secretory and contractile myofibroblasts is a key event that drives the fibrotic response (Kong et al., 2014).

### Epicardium as the Major Source of ECM-Producing Cells

Lineage tracing studies revealed that the prominent source of cFBs, including those activated as response following injury, is a subset of cells originating from the embryonic epicardium (Russell et al., 2011; Zhou et al., 2011, 2012; van Wijk et al., 2012). These cells undergo EMT and migrate into the myocardial wall (Zhou et al., 2008; Ali et al., 2014; Moore-Morris et al., 2014; Hortells et al., 2019; Quijada et al., 2020) as reveled by basic helix–loop–helix (bHLH) transcription factor 21 (Tcf21) (Acharya et al., 2012), T-box transcription factor 18 (Tbx18) (Cai et al., 2008), and Wilms’ tumor 1 (Wt1) (Zhou et al., 2008) reporter mouse lines (Pérez-Pomares et al., 2002; Braitsch et al., 2012; Greulich et al., 2012; Braitsch and Yutzey, 2013). These transcription factors repress genes encoding epithelial adhesion molecules (E-cadherin, claudins, and occludens) and the activation of mesenchymal genes (N-cadherin, collagens, and FN) necessary for ECM production and cell migration (Lamouille et al., 2014; Quijada et al., 2020). This transdifferentiation process is also relying on TGF-β, BMP, Wingless-related integration site (Wnt), and retinoic acid (RA) signaling, reviewed in detail elsewhere (von Gise and Pu, 2012; Braitsch and Yutzey, 2013).

After development, the epicardium becomes relatively dormant; however, despite the differences in duration of regeneration and the nature of the specific injury insult, reactivation of embryonic epicardial potential is conserved in zebrafish and neonatal mouse heart regeneration (Lepilina et al., 2006; Jopling et al., 2010; Kikuchi et al., 2010, 2011b; Chablais et al., 2011; González-Rosa et al., 2011, 2012; Porrello et al., 2011; Schnabel et al., 2011; Wang et al., 2011; González-Rosa and Mercader, 2012; Mercer et al., 2013). Similarly, studies using mouse models of cardiovascular disease and human diseased hearts show that the regulatory programs that promote the cFB lineage development are reactivated in the adult cardiac fibrotic response (Zhou et al., 2011; Braitsch et al., 2013). Although full recapitulation of the embryonic program has not been definitively established, several observations point toward at least some degree of epicardial involvement post injury (Lepilina et al., 2006; Kikuchi et al., 2010, 2011b; González-Rosa et al., 2011, 2012; Porrello et al., 2011; Schnabel et al., 2011; Wang et al., 2011; Jesty et al., 2012; Smits and Riley, 2014).

This way, Tcf21, Tbx18, and Wt1 serve as markers of both developmental and injury-induced epicardial-derived fibroblasts in zebrafish and mammalian adult hearts (Kikuchi et al., 2011a; Braitsch et al., 2013; Ali et al., 2014; Moore-Morris et al., 2014; Kanisicak et al., 2016). Resident cardiac mouse fibroblasts labeled with Col1a1-GFP, PDGFRα, and Tcf21 transgenic alleles display the ability to proliferate after injury and give rise to a majority of cells in the fibrotic scar (Acharya et al., 2012; Kanisicak et al., 2016). Myofibroblasts, labeled by periostin, emerge from Tcf21 lineage-traced epicardium-derived fibroblasts when mice are subjected to MI or left ventricle pressure overload and are a major source of ECM (Kanisicak et al., 2016; Fu et al., 2018).

The epicardium responds to ischemic injury (e.g., MI) through major signaling pathways. Among them, TGF-β/Smad3 is the key intracellular pathway promoting cell activation, namely, fibroblasts, and fibrogenesis (Travers et al., 2016; Table 1). Wnt signaling is activated by the expression of Wnt1 by epicardial cells upon ischemia reperfusion damage *in vivo*, in a mouse model, and *in vitro*, epicardial cells undergo EMT and adopt a fibroblast-like phenotype when treated with Wnt1 (Duan et al., 2012). Crossing WT1^Cre^ with βcatenin^flox/flox^ mice specifically abrogated Wnt signaling in epicardial cells, and as a result, there were minimal expansion of the epicardium post ischemia reperfusion injury and reduced collagen deposition in the subepicardium (Duan et al., 2012). Hippo signaling, which normally keeps cFBs in the resting state, is inactivated after cardiac injury, resulting in spontaneous transition toward a myofibroblast state that favors fibrosis and remodeling (Liu et al., 2015; Piersma et al., 2015; Ramjee et al., 2017; Johansen and Molkentin, 2019). Also involved in the postnatal epicardial response to the ischemic stress is Notch signaling, by modulating the differentiation of profibrotic myofibroblasts and thus counteracting the effects of the profibrotic TGF-β (Ali et al., 2014; Nistri et al., 2017).

### ECM Dynamics in Cardiac Fibrosis

**FIGURE 2:**
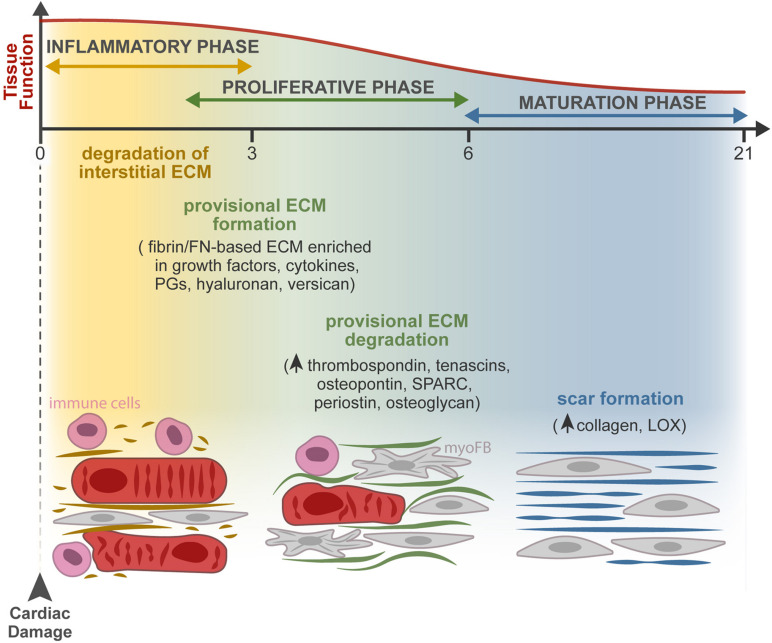
ECM dynamics during tissue repair after MI. Progressive changes in the composition of the ECM occur during the three overlapping phases of the injury response: inflammatory, proliferative, and maturation phases. At the extracellular space, the main remodeling events encompass the degradation of the interstitial matrix (dark yellow lines), production and resolution of the provisional ECM (green lines), and lastly, scar formation (blue lines). Firstly, the release of inflammatory mediators by dead cells leads to the recruitment of leukocytes and neutrophil activation (pink cells) and increases vascular permeability and MMP expression and activity. The latter degrades the interstitial matrix (yellow), generating bioactive fragments (matrikines) that contribute to the inflammatory cascade. From the extravasated plasma proteins, a fibrin- and FN-based matrix network is formed (provisional ECM, green). This transient ECM is rich in growth factors and inflammatory cytokines and serves as a highly permeable conduit for cells. Fibroblasts (gray cells) adhere to this matrix, initiate the repair of the damaged area through proliferation and differentiation in myofibroblasts (myoFBs), and secrete different ECM molecules, such as proteoglycans (PGs), hyaluronan, and versican, that stabilize this provisional ECM. During the proliferative phase, myoFBs deposit large amounts of structural ECM proteins, mostly collagens, to preserve the integrity of the myocardial wall, and the provisional matrix is degraded. As the maturation phase initiates, the collagen content increases, and enzymes such as LOX are upregulated, inducing collagen cross-linking and the formation of a rigid scar.

Following cardiomyocyte death, subjacent to cardiac insult, dynamic changes in the composition of the ECM act as regulators of the cellular responses leading to cardiac repair (Dobaczewski et al., 2010). The repair process can be divided into three overlapping phases: an inflammatory phase, a proliferative phase, and a maturation phase (Figure 2). At the extracellular space, four key events occur during repair, namely, the degradation of the interstitial matrix, production and resolution of the provisional ECM, and lastly, scar formation.

The death of cardiomyocytes after MI triggers an inflammatory reaction through the release of inflammatory mediators (cytokines and chemokines) that leads to the recruitment of leukocytes and neutrophil activation, revised in detail by Ong et al. (2018). Inflammation increases vascular permeability, resulting in extravasation of plasma proteins like fibrin, fibrinogen, and FN, and increases MMP expression and activity, leading to degradation of the interstitial matrix generating bioactive fragments (matrikines) that contribute to the activation of the inflammatory cascades. Consequent formation of a fibrin- and FN-based matrix network formed from the extravasated plasma proteins, known as provisional ECM, enriched with growth factors (PDGF, FGF, VEGF, and TGF families) and inflammatory cytokines secreted by various cell types, serves as a highly permeable conduit for infiltrating inflammatory cells (Dobaczewski et al., 2006; Bujak et al., 2008; Saxena et al., 2013; Takawale et al., 2015; Barker and Engler, 2017; Frangogiannis, 2017). Fibroblasts and other resident cells can adhere to this matrix, enabling fibroblast migration and inducing fibroblast proliferation and transdifferentiation to start the repair of the damaged areas (Serini et al., 1998; Rybarczyk et al., 2003; Chistiakov et al., 2016). Fibroblasts in the provisional ECM secrete other ECM molecules, such as proteoglycans, hyaluronan, and versican, that stabilize this provisional matrix (Wight and Potter-Perigo, 2011). Clearance of dead cells and ECM debris by phagocytes induces the release of anti-inflammatory mediators necessary for the resolution of the inflammatory phase, marking the transition to the proliferative phase. At this point, the ECM is enriched with matricellular proteins that modulate cellular phenotype, activate proteases and growth factors, and impinge on signaling cascades (Murphy-Ullrich and Sage, 2014). During the proliferative phase, growth factors secreted by mononuclear cells and macrophages activate myofibroblast-mediated deposition of large amounts of structural ECM proteins. The provisional matrix is degraded, and cellular FN is secreted primarily by fibroblasts and macrophages. Cellular FN containing extra domain A together with TGF-β and mechanical tension were required for myofibroblast transdifferentiation (Hinz et al., 2007; Shu and Lovicu, 2017). ECM structural proteins are then deposited to preserve the integrity of the myocardial wall (Zymek et al., 2006; Nielsen et al., 2019). While most matricellular proteins are rare or absent in the healthy myocardium, they are highly upregulated following cardiac injury. These proteins do not play a structural role but modulate cell function, promote matrix assembly, and protect the myocardium from adverse remodeling, as reviewed in Frangogiannis (2017). They include TSPs, TNC and TNX, osteopontin (OPN), secreted protein acidic and cysteine rich (SPARC), periostin, osteoglycin, and members of the cellular communication network factor (CCN) family (Dobaczewski et al., 2010; Kong et al., 2014). Recently, a different role of myofibroblasts has been described, as these cells were found capable of engulfing dead cells and acquiring an anti-inflammatory phenotype. The findings show that myofibroblasts cooperate with infiltrating macrophages to remove dead cells, raising the hypothesis that myofibroblast-mediated engulfment may itself activate the production of ECM proteins independently of macrophages (Nakaya et al., 2017).

Some studies suggest that the end of the proliferative phase and beginning of the maturation phase are marked by apoptosis of the majority of the myofibroblasts—to eliminate the granulation tissue cells from the infarcted area—however, the mechanism behind this proapoptotic process has not been fully investigated (Zhao et al., 2004; Xue and Jackson, 2015). The collagen content increases, and the upregulation of enzymes such as LOX induces collagen cross-linking (Al-U’datt et al., 2019). Therefore, a rigid scar is formed without contractility and relaxation capacity, ultimately leading to heart stiffening, electrical signaling impairment, and consequent heart failure (Miragoli et al., 2007; Richardson et al., 2015; Murtha et al., 2017). The existence of endogenous mechanisms that restrain the matricellular signals to protect the myocardium from progressive fibrosis, when a mature ECM environment is formed, remains to be explored.

## ECM Modulation

Despite the advances on the role of the ECM in cardiac pathophysiology, the mechanisms that drive the feedback communication between ECM remodeling and cell response are not fully elucidated due to their intricate nature. Hence, the establishment of *ex vivo* model systems replicating the native myocardium is central to address further fundamental mechanistic questions.

### Decellularization as a Methodology to Deconstruct ECM Composition, Structure, and Bioactivity

**FIGURE 3:**
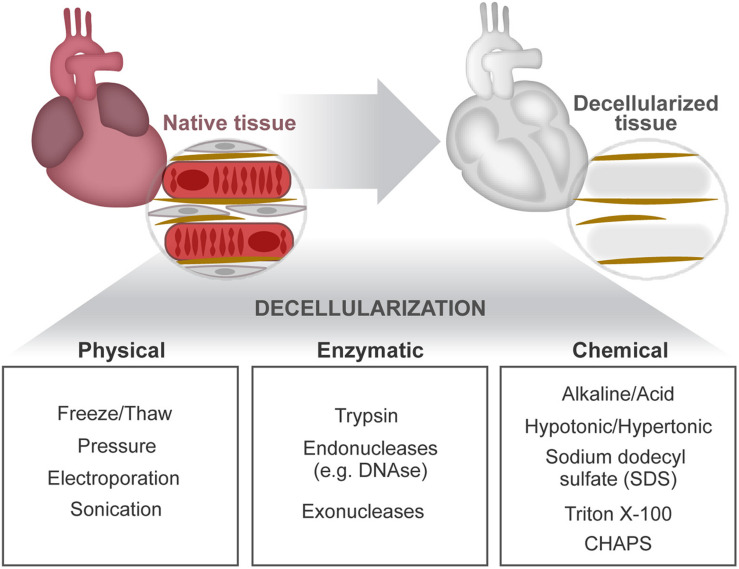
Cardiac decellularization. Decellularization aims to remove the cellular compartment of a tissue, while preserving the composition and architectural arrangement of the ECM. This can be achieved by combined application of physical, enzymatic, and chemical treatments.

First, insights on the relevance of cell–ECM cross talk in the heart came forth through the observation of two-dimensional (2D) immunostainings of tissue sections and the analysis of pathophysiological alterations resultant of mutations in ECM-related genes or perturbations in related signaling pathways. The development of decellularization methodologies facilitated further structural (Silva et al., 2016) and proteomic analyses (e.g., mass spectrometry) of the extracellular contents (Mayorca-Guiliani et al., 2017) by providing ECM protein enrichment as a result of the cellular content removal (cellular “noise”) (de Castro Bras et al., 2013; Naba et al., 2015; Silva et al., 2016). Decellularization separates tissue ECM by the removal of cells and their associated material. This is achieved by applying, alone or in combination, chemical (buffers and detergents), enzymatic (trypsin and DNase), and physical (agitation and sonication) agents delivered using different techniques (perfusion, immersion, and agitation) (Beacham et al., 2007; Ott et al., 2008; Badylak et al., 2011; Crapo et al., 2011; Gillies et al., 2011; Figure 3). Ultimately, the central goal of decellularization is to obtain a balance between clearance of cellular materials and the retention of a close-to-native ECM.

Sodium dodecyl sulfate (SDS) is the most common detergent used for cardiac tissue decellularization. SDS concentration and duration of exposure affects greatly the preservation and integrity of the ECM. High SDS concentrations induce protein denaturation, collagen fibril disruption, and removal of GAGs (Gilbert et al., 2006; Crapo et al., 2011). In contrast, low SDS concentrations have been shown to preserve important ECM features, such as coil structures identified on fetal mouse heart ECM (Silva et al., 2016). Hence, reliable comparisons of different ECM microenvironments require the use of similar preparation methods. A versatile decellularization protocol working efficiently on distinct organs and also on the same tissue but on different ontogenetic stages (young and adult tissues) or health status has been recently reported (Silva et al., 2016, 2019; Garlikova et al., 2017; Pinto et al., 2017). This approach mitigates differences resultant from the application of distinct decellularization protocols, allowing fair comparisons on ECM composition and functional alterations across tissues, age, and normalcy vs. disease status (Perestrelo et al., 2020).

Decellularization can be performed on tissues/organs or on cells cultured *in vitro* as monolayers or aggregates (Beacham et al., 2007; Nair et al., 2008). The latter *in vitro* models facilitate manipulation of ECM-related genes (e.g., gene knockdown) to expose the role of specific ECM molecules (Ott et al., 2008; Williams et al., 2014; Silva et al., 2016; Pinto et al., 2017). For instance, Kong et al. (2018) using different approaches, including CRISPR/Cas9-mediated knockout of hyaluronan synthase 2 (the enzyme necessary to produce hyaluronan), found that hyaluronan inhibits vascular calcification involving BMP2 signaling. Despite being straightforwardly obtained and manipulated, *in vitro*-derived ECM misses to recreate the native organ ECM complexity. To the contrary, tissue-derived ECM often preserves native biochemical and mechanical properties, constituting an attractive alternative for studying the impact of ECM on complex scenarios such as age, disease, and injury as well as for therapeutic applications in regenerative medicine. Decellularized tissues were readily translated into the clinic as surgical scaffolds since ECM molecules are highly preserved across species, permitting the application of allogenic and xenogenic tissue-derived ECM. These applications demonstrated low immunogenicity while promoting specific cell functions (Wicha et al., 1982; Badylak, 2004; Gilbert et al., 2006; Crapo et al., 2011; Svystonyuk et al., 2020). Decellularization methodologies have evolved toward whole-organ decellularization by improvements such as the delivery of decellularization agents through the vasculature (perfusion) which promotes clearance of cellular remnants *in situ* (Ott et al., 2008; Badylak et al., 2011). Nevertheless, tissue-derived ECM holds limitations related both to batch-to-batch variability and to contaminants remaining after ineffective cell removal.

The development of decellularization methods opened new avenues to a more detailed assessment of tissue-derived ECM and of *in vitro* ECM–cell interactions. This will ultimately lead the way to the development of ECM-based therapies.

### Cardiac ECM-Based Strategies for Regeneration and Repair

Excessive ECM, common to several cardiac pathologies, is an obstacle for normal organ function (Richardson et al., 2015), and clinical therapeutic strategies to control cardiac fibrosis are still on the horizon (Tzahor and Poss, 2017).

Previously reported ECM-derived therapies for MI encompass: (i) the delivery of decellularized cardiac ECM (Johnson et al., 2014; Wang et al., 2019), (ii) scaffolds functionalized with ECM-derived proteins or peptides (Zhang et al., 2019), and (iii) biomaterials that mimic the ECM (Youngblood et al., 2018; Yuan et al., 2019) and that are able to deliver soluble cytokines/growth factors (Rufaihah et al., 2017), miRNAs (Bheri and Davis, 2019), or cells (Chakravarti et al., 2018).

Cardiac-derived ECM, obtained by decellularization, has the benefit of conserving the organ-specific ECM architecture and composition and ensuing retention of biochemical cues that favor recellularization (Kc et al., 2019) and has shown promising results in animal models (Wainwright et al., 2012; Sarig et al., 2016; Wang et al., 2016) and for human applications (Rego et al., 2019). However, decellularized matrices still pose many technical challenges that need to be addressed to meet clinical standards (Kc et al., 2019). Unfortunately and due to the complex composition of tissue-derived ECM, most authors do not attempt to discriminate which ECM factors or properties (e.g., architecture and stiffness) contribute to the observed beneficial effect.

Our growing knowledge on cardiac ECM function paves the way for new and promising therapeutic targets that can not only repair the injured heart but also induce cardiac regeneration. Recently, peptides generated from the degradation of ECM proteins have gained increasing attention for therapeutic application as increasing evidence supports that these molecules regulate various processes during cardiac repair and homeostasis (Ricard-Blum and Vallet, 2019). For example, p1158/p1159, the products of MMP2- and MMP9-mediated degradation of type I collagen, has been shown to promote angiogenesis and to reduce scar formation after MI (Lindsey et al., 2015). Canstatin, the product of MMP2-mediated degradation of type IV collagen, has been shown to regulate cardiomyocyte calcium channel activity (Imoto et al., 2018) and to reduce hypoxia-induced cardiomyocyte apoptosis (Okada et al., 2016). Tumstatin, the product of MMP9-mediated degradation of type IV collagen, protects cardiomyocytes against reactive oxygen species (ROS)-induced apoptosis (Yasuda et al., 2017), and on the other hand, endostatin, a cleaved fragment of type XVIII collagen, increases the proliferation and migration of myofibroblasts (Sugiyama et al., 2018).

The extracellular proteins from the SPARC family have important roles in cellular adhesion, migration, and proliferation modulating ECM processing and the TGF-β signaling (Bradshaw, 2012). Follistatin-like 1 (FSTL1), for instance, a member of this family, is a BMP4 antagonist that can improve heart function after MI (Altekoester and Harvey, 2015) and abrogates aldosterone-induced cardiac myocyte hypertrophy (Tanaka et al., 2016). However, only recombinant FSTL1 produced in bacteria or epicardium-derived, but not myocardium-derived, FSTL1 activates cardiomyocyte proliferation and cardiac regeneration (Wei et al., 2015). This appears to relate with the glycosylation of FSTL1 since a single replacement of asparagine with glutamine in the N-glycosylation site at position 180 of human FSTL1, hampering glycosylation at this position, was enough to activate cardiomyocyte proliferation and limit cardiac remodeling post MI, following the delivery of this modified FSTL1 mRNA to the mouse myocardium (Magadum et al., 2018).

Another relevant function of the ECM is to work as a reservoir of bioactive molecules, namely, miRNAs. The latter are also able to modulate cardiomyocyte proliferation and cardiac repair (Katz et al., 2016). For example, miR-17–miR-92, miR-199a, miR-214, miR-222, miR-302–miR-367, and miR-590 can promote cardiomyocyte proliferation and cardiac regeneration, whereas miR-15 family miRNAs inhibit cardiomyocyte proliferation and cardiac repair (Hashimoto et al., 2018; Deshmukh et al., 2019).

An emergent area in cardiac ECM for therapeutic purposes is the exploitation of young ECM as a source of regenerative targets as different findings support that severe changes in the ECM and in fibroblasts may dictate the loss of cardiac regenerative capacity after birth (Notari et al., 2018; Hortells et al., 2019). In fact, neonatal cardiac ECM improves myocardial function *in vivo*, reduces MI-induced fibrosis, and promotes angiogenesis and endothelial cell activity while the adult counterpart showed no beneficial effect (Wang et al., 2019). Consistently, the dystrophin complex protein agrin, whose expression in the heart decreases from P1 to P7, is an important regulator of cardiomyocyte division during the transient neonatal regenerative period (Bassat et al., 2017). Conditional deletion of *Agrn* in the cardiac mesoderm promoted maturation and reduced cell cycle activity of cardiomyocytes and impaired cardiac regeneration at P1 (Bassat et al., 2017). Bassat et al. (2017) also showed that intramyocardial administration of recombinant agrin after MI in a mouse model promotes moderate cardiomyocyte cell cycle reentry and proliferation on the healthy heart near the injury, leading to a significant reduction of the scar area 35 days after MI and improved cardiac function, when compared with the control. In an *in vitro* setting, agrin promoted proliferation and delayed maturation of induced pluripotent stem cell-derived cardiomyocytes (iPSC-CM) through Dag1, extracellular signal-regulated kinase (ERK), and YAP signaling. Another example of an ECM-associated protein highly expressed in the postnatal heart and barely detectable in the adult heart is periostin (Snider et al., 2008). The latter promotes cardiac regeneration by switching differentiated cardiomyocytes into cycling cells, improving cardiac function after MI (Kühn et al., 2007). However, whereas periostin-knockout mice showed impaired regeneration and abundant fibrosis following MI at P1 (Chen et al., 2017), no effect was reported for periostin knockout or overexpression on cardiomyocyte proliferation after MI in adult mice (Lorts et al., 2009). In fact, periostin also regulates cardiac fibrogenesis as targeted ablation of fibroblasts expressing periostin precludes adverse cardiac remodeling (Kaur et al., 2016). This exemplifies how the pleiotropic effect of different ECM proteins may complicate their direct application for therapeutic purposes.

## Conclusion

**FIGURE 4:**
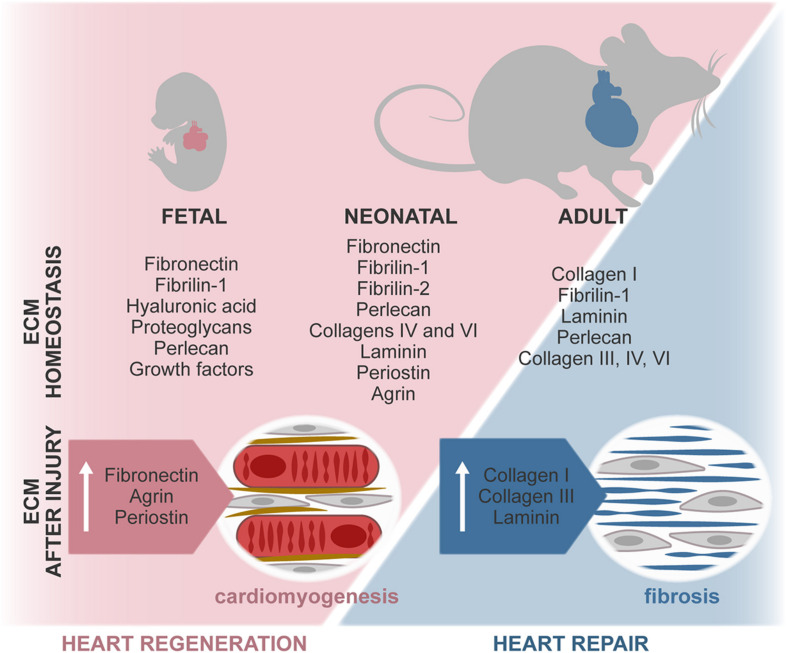
ECM composition during regenerative (fetal/neonate) and reparative (adult) stages. The composition of the ECM changes around birth, resulting in a stiffer and less regenerative environment. In the fetal–neonatal heart, agrin and periostin stimulate cardiomyocyte proliferation and neovascularization, thus promoting regeneration of the tissue. During adult heart repair, increased expression of fetal-associated ECM is observed, namely, through the expression of FN and hyaluronan. However, this reactivation of the fetal program is incomplete, and adult cardiomyocytes are unable to proliferate, resulting in the formation of a collagen-rich scar.

Regulation of heart formation, homeostasis, and response to injury derives from intricate interactions between cells and their extracellular microenvironment. A misbalance on the expression of ECM and ECM-related molecules often leads to congenital malformations and development of disease. Although *in vitro* studies have exposed the relevance of several microenvironmental features, the dynamics of the complex 3D ECM network throughout life and its effect on cardiac cells remain largely elusive. Recently, different studies revealed that ECM-associated factors promote neonatal heart regeneration and that changes on ECM stiffness may limit this capacity to the first days after birth (Figure 4). These evidences, together with *in vitro* studies showing the beneficial properties of young ECM on cardiac cells, support that tissue engineering and regenerative medicine strategies aimed at promoting cardiac regeneration could benefit from mimicking the fetal–neonatal extracellular environment.

## Author Contributions

All authors designed, drafted, and revised the manuscript.

## Conflict of Interest

The authors declare that the research was conducted in the absence of any commercial or financial relationships that could be construed as a potential conflict of interest.

## Abbreviations

ADAMTs: A disintegrin and metalloproteinase with thrombospondin repeats
AV: Atrioventricular
BMP: Bone morphogenetic protein
cFB: Cardiac fibroblasts
ECM: Extracellular matrix
EndoMT: Endocardial-to-mesenchymal transition
EMT: Epithelial-to-mesenchymal transition
E: Embryonic day
FN: Fibronectin
FSTL1: Follistatin-like 1
GAGs: Glycosaminoglycans
HA: Hyaluronic acid
LOX: Lysyl oxidase
MI: Myocardial infarction
miRNAs: MicroRNAs
MMPs: Matrix metalloproteinases
NCCs: Neural crest cells
P: Postnatal day
SDS: Sodium dodecyl sulfate
SPARC: Secreted protein acidic and cysteine rich
TAZ: Transcriptional coactivator WWTR1
Tbx18: T-box transcription factor 18
Tcf21: Transcription factor 21
TGF- β: Transforming growth factor β
TNC: Tenascin-C
TSP: Thrombospondin
YAP: Yes-associated protein
Wnt: Wingless-related integration site
Wt1: Wilms’ tumor 1
3D: Three-dimensional.
